# Tuberculosis in healthcare workers – a narrative review from a German perspective

**DOI:** 10.1186/1745-6673-9-9

**Published:** 2014-03-14

**Authors:** Albert Nienhaus, Anja Schablon, Alexandra M Preisser, Felix C Ringshausen, Roland Diel

**Affiliations:** 1 Institute for Health Service Research in Dermatology and Nursing (IVDP), Center of Excellence for Epidemiology and Health Service Research for Healthcare Professionals (CVcare), University Medical Center Hamburg-Eppendorf, Martinistraße 52, Hamburg 20246, Germany; 2 Principles of Prevention and Rehabilitation Department (GPR), Institute for Statutory Accident Insurance and Prevention in the Health and Welfare Services (BGW), Hamburg, Germany; 3 Institute for Occupational and Maritime Medicine, University Medical Center Hamburg-Eppendorf (UKE), Hamburg, Germany; 4 Department of Respiratory Medicine, Hanover Medical School, Hanover, Germany; 5 Institute of Epidemiology, University Medical Center Schleswig-Holstein (UKSH), Kiel Campus, Kiel, Germany; 6 Lung Clinic Grosshansdorf, Member of the German Center for Lung Research (DZL), Grosshansdorf, Germany

**Keywords:** Tuberculosis, Provision, Occupational disease, Assessment, Healthcare, Prevention

## Abstract

**Introduction:**

Despite the decline of tuberculosis in the population at large, healthcare workers (HCW) are still at risk of infection.

**Methods:**

In a narrative review the TB risk in HCW and preventive measures are described, with the focus on epidemiology and Occupational Safety and Health (OSH) regulations in Germany.

**Results:**

There is an increased risk of infection not only in pneumology and laboratories with regular contact with tuberculosis patients or infectious materials. Epidemiological studies have also verified an increased risk of infection from activities that involve close contact with patients’ breath (e.g. bronchoscopy, intubation) or close contact with patients in need of care in geriatric medicine or geriatric nursing. In occupational disease claim proceedings on account of tuberculosis, the burden of proof can be eased for insured persons who work in these or other comparable fields. Forgoing evidence of an index person as a source of infection has led to a doubling of the rate of cases of tuberculosis recognised as an occupational disease and has halved the duration of occupational disease claim proceedings in Germany. For several years now, it has been possible to use the new interferon-y release assays (IGRAs) to diagnose a latent tuberculosis infection (LTBI) with significantly greater validity than with the traditional tuberculin skin test (TST). However, variability of the IGRAs around the cut-off poses problems especially in serial testing of HCWs. At around 10%, LTBI prevalence in German healthcare workers is lower than had been assumed. It can make sense to treat a recent LTBI in a young healthcare worker so as to prevent progression into active tuberculosis. If the LTBI is occupational in origin, the provider of statutory accident insurance can cover the costs of preventive treatment. However, little is known about disease progression in HCWs with positive IGRA sofar.

**Conclusion:**

TB screening in HCWs will remain an important issue in the near future even in low incidence, high income countries, as active TB in HCWs is often due to workplace exposure. The IGRAs facilitate these screenings. However, variability of IGRA results in serial testing of HCWs need further investigations.

## Introduction

Tuberculosis (TB) is the second most frequent work-related infectious disease among German healthcare workers (HCW) [1]. Understanding the dynamics of infection risks for HCWs and developing an adequate Occupational Safety and Health (OSH) system is crucial to protect HCW from TB and to limit the spread of infections from HCWs to patients. In this narrative review we describe the German perspective and experience with TB in HCWs. As Germany has an elaborate system for occupational disease prevention and rehabilitation, telling the story from a German perspective might encourage other countries with less developed OSH systems [2] to improve infection control in HCWs.

### Epidemiology of tuberculosis in Germany

**Figure 1:**
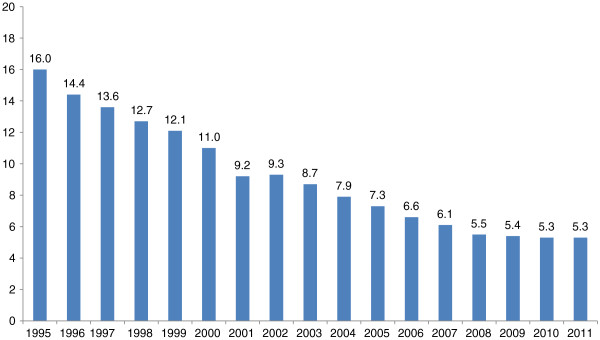
**Incidence of TB in Germany in the years 2000 to 2011 **[3]**.**

According to information provided by the Robert Koch Institute (RKI), in 2011 a total of 4,317 cases of tuberculosis were registered in Germany (previous year: 4,388), equivalent to an incidence of 5.3 (previous year: 5.3) new cases per 100,000 inhabitants [3]. The clear downward trend observed in Germany in recent years thus almost ground to a halt (Figure 1). The lung was the organ most frequently affected by TB, accounting 79.6% (3,346) of cases. Around one third (33.9%) of lung tuberculosis were of the infectious, microscopically positive type. The success of treatment in higher age groups declined continuously to just 63.3% in patients aged 70 and over. The incidence of TB among citizens with a migration background is approximately four times higher than among German nationals (5.3 versus 21.5/100,000).

### Transmission paths and clinical picture of TB

Bacteria of the *Mycobacterium tuberculosis* (MTB) complex are transmitted from person to person by droplet infection and rarely via smear infections on skin and mucous membranes, via contaminated dust particles or cuts and stab wounds involving contaminated cannulae or scalpels [4]. Environmental factors, hygiene conditions, the amount of germs discharged and the virulence of the pathogens determine the degree of infection risk. Personal factors such as nutritional condition, age, immune status and comorbidity determine susceptibility of infection and the progression of the disease.

### Primary tuberculosis or latent TB infection (LTBI)

The risk of infection increases with the duration and closeness of contact with the source of infection. However, brief occasional contacts – even a single conversational contact – can also lead to infection [5]. After an incubation period of five to six weeks on average (maximum 8 weeks), an inflammatory reaction occurs in the lung (primary focus) and possibly in the regional lymph nodes (primary complex). In most cases, this process heals without significant clinical symptoms. Sometimes calcification takes place, so that the primary focus and possibly also the associated lymph nodes remain radiologically identifiable at a later date (Ghon foci). However, it is also possible that active tuberculosis will develop immediately, with a progressive, infiltrative and cavitating process and/or with bronchogenic, haematogenous or lymphogenous spread, leading to further organ manifestations.

In the primary complex, which often cannot be rendered visible, viable mycobacteria capable of replication and proliferation may remain for years or decades. This leads to sensitisation of monocytes circulating in the peripheral blood. The presence of these sensitised monocytes can be measured by means of a tuberculin skin test (TST) or with the interferon-y release assays (IGRAs) that have recently become available. Both tests are based on the cell-mediated immune response to *M. tuberculosis* antigens. However, the IGRA is considerably more specific than the TST, since it works with only two or three *M. tuberculosis* antigens instead of approximately 200 antigens as in the tuberculin for the TST [6,7]. Therefore, a positive TST should be validated by an IGRA so as to rule out non-specific reactions, e.g. to a Bacillus Calmette-Guerin (BCG) vaccination or to environmental non-tuberculous mycobacteria (NTM). Two different IGRAs are commercially available, the ELISA-based QuantiFERON-TB Gold In-Tube^®^ and the ELISPOT-based T-SPOT.TB*^®^*.

Since IGRAs are *in vitro* tests, the problem of boosting that occurs in serial testing with TST is avoided. The IGRA correlates better than the TST with exposure to infectious patients and shows higher sensitivity to active TB than the TST [6-9]. Moreover, IGRAs have a higher predictive value in contact tracings of persons with close contact to infectious patients in low-incidence countries as regards the progression of the disease [10,11]. Even though only one study reported progression risk in HCWs based on four cases [11], IGRAs are likely to improve both the effectiveness and the efficiency of TB screening in HCWs [12]. However, as yet no consensus exists on the interpretation of IGRA results in serial testing [6,7,13]. In serial testing especially, it is important to state IGRA results not only qualitatively but also quantitatively.

Some more water needs to be purred into the wine. As IGRA are not able to distinguish LTBI from active TB, IGRA are no reliable test for the diagnosis of active TB [14].

A conversion – when a previously negative test result becomes positive when measured again – indicates a fresh infection with *M. tuberculosis*. Reversion of immunological tests from positive to negative is possible if MTB is successfully eliminated (transient infection) or if there is so little MTB activity in the primary complex that T-cells are no longer sensitised [15].

### Post-primary tuberculosis

In the generalisation phase, the mycobacteria spread through the blood or lymph, developing a focus in the lung or other organs. Initially, this is usually silent. If such foci are reactivated – in the lungs, usually in the apex area – active tuberculosis of the lung, or for example of the kidney, genitals or bone, may develop months or even decades later, requiring treatment. As mentioned above, around one-third of new cases are extra-pulmonary [3]. These forms of tuberculosis result from the reactivation of primary tuberculosis, or may be caused by re-infection.

### Diagnosis

Diagnosing TB is sometimes difficult due to the diverse forms of manifestation. In addition, diagnosis is often belated because there are no specific symptoms and the possibility of TB is too rarely considered in differential diagnosis.

Symptoms or circumstances that indicate the presence of TB are:

• Clinical symptoms such as coughs, phlegm, weight loss, tiredness, nocturnal sweating, fever, breathing-dependent pain in the thorax

• Tuberculosis in the anamnesis

• Occupational or private exposure to tuberculosis

• Positive interferon-y release assay (IGRA)

• X-ray results raising suspicion of tuberculosis

The diagnosis is confirmed by evidence of tuberculosis pathogens in test materials such as sputum, bronchial secretions, gastric juice, urine or pus from an abscess. The quickest method is microscopic examination. However, this is crude and only shows positive results at approx. 10^4^–10^5^ bacteria/ml of material. Since only acid-fast bacilli (AFB) can be verified under the microscope, the diagnosis of TB is not confirmed. Only a positive culture confirms TB. Cultural verification is possible with only around 50–100 bacteria/ml in the test material. Alternatively, TB can be confirmed by molecular biology methods (e.g. polymerase chain reaction, PCR) to verify TB-specific DNA or RNA.

Welcome progress has been made in developing these methods further in recent years. In an attempt to create an easy-to-use, low-cost test, the Xpert MTB/RIF system has become prevalent. The WHO has been recommending the use of the test since 2010 for initial diagnosis where tuberculosis is suspected, primarily in high-prevalence countries with a high proportion of HIV patients and where multi-drug-resistant (MDR) tuberculosis is suspected. The Xpert MTB/RIF offers a fully automatic real-time PCR for *M. tuberculosis* complex-specific DNA and a sub-unit of bacterial RNA polymerase beta (rpoB), which is observed in 95% of resistance to Rifampicin. It is easy to handle and takes about 20 minutes of active work, with a result available in two hours. Admittedly, the Xpert MTB/RIF is not sensitive enough to be the sole method used in developed countries. The role of Xpert MTB/RIF in the diagnosis of TB in high income, low TB incidence countries is currently under study [16].

### Risk factors for tuberculosis

The WHO assumes that 30% of the world’s population is infected with *M. tuberculosis*. These latent TB infections (LTBIs) lead to active TB in 10% of those affected during the course of their lives. In around half, active TB develops within the first two years after infection. Children are more likely than adults to develop active TB after infection (around 30%) [10]. Malnutrition, homelessness and alcoholism, along with HIV infection and other diseases that weaken the immune system, or immunosuppressive therapies (in which TNF-alpha blockers are especially significant) are typical risk factors for activation of LTBI.

It was previously assumed that in countries with a low incidence of TB most cases of active tuberculosis result from reactivation of an LTBI. However, infection epidemiology findings from cluster analyses using fingerprinting to differentiate between strains suggest that new infections in developed countries may account for up to 40% of active tuberculosis cases [17,18], rather than 10% as had been previously assumed [19]. An LTBI or active TB in the anamnesis does not protect against reinfection. Depending on the country and study, reinfections are responsible for 10% to 75% of all second episodes of tuberculosis [20]. However, it can also be shown that targeted prevention measures can significantly reduce transmissions of *M. tuberculosis*[21,22].

### Tuberculosis risks in healthcare

Healthcare workers are at increased risk of LTBI and active tuberculosis [22-25]. However, in countries with a low incidence of TB, it can be difficult to verify this increased risk of infection since the customary risk factors for both infection and for active TB are less prevalent than among the general population, and not all healthcare workers have contact with TB patients. Moreover, appropriate preventive action (see below) can be taken to minimise the risk of infection when caring for patients known to be suffering from tuberculosis [24].

**Figure 2:**
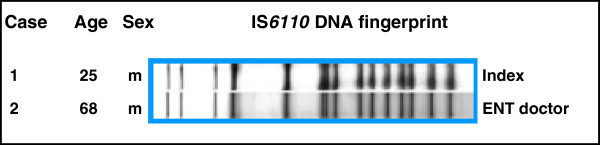
**Example of two identical fingerprints from the Hamburg fingerprint study **[4]**.**

Fingerprinting and the supplementary molecular biology methods for differentiating between strains of *M. tuberculosis* improve the possibility of discovering infection pathways. Individuals with different strains of TB cannot have infected each other. Individuals with the same strain of TB may be part of a common infection pathway. In molecular epidemiology, patients with the same strain of TB are known as clusters (Figure 2). However, belonging to a cluster cannot be equated with confirmed transmission of TB. Rather, an appropriate infectious contact needs to be ascertained as probable by interviewing the members of the cluster. Three molecular epidemiology studies on the occupational risk of infection have been published so far [4,26,27].

In the Hamburg fingerprint study [4], mycobacteria cultures have been used continuously since 1997 to generate fingerprints of all individuals with notifiable TB. The study covers 848 cases where IS6110 fingerprinting was carried out. Ten study participants with TB were healthcare workers. Through fingerprinting and a subsequent survey, it was possible to prove occupation-related transmission in eight of those individuals. Thus the proportion of occupation-related tuberculosis in healthcare workers was 80% (95%CI 44%–97%). Of eight occupation-related transmissions, six were unknown prior to fingerprinting.

A fingerprint study on the infection risk among healthcare workers in the Netherlands found that in 28 out of 67 (43%) persons with active TB, transmission from patient to staff was proved [26]. The main reason for transmission was belated diagnosis of index cases, especially among older patients. Ten (out of 28) index cases were older people with comorbidity whose tuberculosis had not been diagnosed promptly, as a result of which no appropriate preventive measures had been taken.

The most up-to-date molecular epidemiology study on the occupational infection risk included all TB cases reported in San Francisco from 1993 to 2003 [27]. In all, there were 2,510 TB cases. Fingerprinting was then performed for 1,852 patients. Thirty-one patients (1.2% of the total) were healthcare workers. Occupationally related TB was confirmed in ten (32%) of the 31 healthcare workers.

### LTBI prevalence in healthcare workers

LTBI is assumed when the IGRA is positive and active TB is excluded by X-ray. In German HCWs LTBI prevalence depends on the age of employees. It increases from 2.6% among the under-25 s to 22.2% among the over-55 s (Table 1) [28-31]. The prevalence of LTBI is the same for men and women. The reason for investigation pursuant to the German regulation on occupational healthcare provisions (ArbMedVV) (see below) also has no influence on the prevalence of LTBI. Likewise, there are no differences between doctors and nurses. LTBI prevalence is higher among geriatric care nurses, otherwise no differences were described between the different occupational groups and activities in Germany [29-31]. About 15% of German HCWs are foreign born and their prevalence of LTBI is more than two times higher than in German born HCWs [30].

**Table 1:** **Latent TB infections (positive IGRA) among healthcare workers according to the German TB network of occupational health physicians, following **[28-31]

**Variable**	**N = 3,823 n (%)**	**IGRA**	
**Age**		**Negative n (%)**	**Positive n (%)**
< 25 years	494 (12.9)	481 (97.4)	13 (2.6)
25-35 years	926 (24.2)	878 (94.8)	48 (5.2)
35-45 years	1,057 (27.6)	982 (92.9)	75 (7.1)
45-55 years	974 (25.5)	867 (89.0)	107 (11.0)
>55 years	372 (9.7)	297(79.8)	75 (20.2)
Gender			
Female	2,959 (77.4)	2,716 (91.8)	243 (8.2)
Male	864 (22.6)	789 (91.3)	75 (8.7)
Reason for screening			
Regular contact with TB patients or infectious material (obligatory)	2,533 (66.3)	2,310 (91.2)	223 (8.8)
Contact tracing (optional)	1,290 (33.7)	1,195 (92.6)	95 (7.4)
Profession			
Physician	583 (15.2)	538 (92.3)	45 (7.7)
Nurse	1,962 (51.3)	1,804 (91.9)	158 (8.1)

LTBI prevalence among healthcare workers in Germany is considerably lower than had been assumed on the basis of studies based on TST [29]. Moreover, the prevalence of LTBI in Germany is significantly lower than in France or Portugal (10%, 19%, 33%) [32,33]. As yet, no comparative data on the prevalence of LTBI in the unaffected population is available for Germany.

### Prevention of tuberculosis

The most effective measures for preventing TB infection are early identification of cases and isolation. It is therefore important for hospitals to consider the possibility of TB when corresponding symptoms arise. The risk of progression from LTBI to active TB can be reduced by preventive chemotherapy [34].

Table 2 summarises the main protective measures for effective control of tuberculosis in the clinical field. Various studies proved that implementing these measures to prevent infection contributes substantially toward reducing the risk for healthcare workers [34-36]. However, it is not possible to make any specific deductions as to the significance of individual sub-aspects. Clearly, organisational and administrative measures are very important, followed by technical and individual preventive measures. Ultimately, only the interaction of various preventive measures can reduce the risk of infection to a minimum.

**Table 2:** **Tuberculosis prevention measures in the inpatient sector, following**[35]

	
Organisational/administrative measures	- Early establishment of a diagnosis
	- Isolation
	- Early initiation of appropriate therapy
Patient	- Information and clarification
	- Cough hygiene
	- Mouth and nose protection
Employee	- General hygiene
	- Respiratory protection
	- If necessary, protective clothing
	- Education and training
	- Operational monitoring
Workplace	- Correct room ventilation
	- Suitable disinfection

### Isolation

The decision whether to isolate must be made on a case-by-case basis. Isolation is indicated as a matter of principle when open, and therefore infectious, tuberculosis of the respiratory tract is suspected or confirmed. The decision whether to isolate patients with negative sputum microscopy must be made individually. Isolation in special tuberculosis wards is not necessary, but the patient should be in a single room. Cohort isolation should only be effected when direct infection chains are assumed (e.g. mother–child). Tests and transfers to other departments for diagnostic or therapeutic purposes that can be postponed should be deferred if possible until the patient can be assumed to be no longer infectious.

As a rule, isolation measures are not necessary in cases of extrapulmonary tuberculosis, though infectious material must be treated with appropriate caution [37].

The decision whether to discontinue isolation depends primarily on the microscopically verifiable decrease in the secretion of pathogens in sputum and on the clinical and radiological response to therapy. Three microscopically negative sputum results are necessary. In most cases, sputum conversion takes place three weeks after the start of an effective therapy. In individual cases (cavernous processes, absence of bacteriological and/or clinical evidence that therapy has been successful, drug resistance, immunosuppression, lack of compliance), however, a longer period of isolation may be necessary. In such cases, the date for ceasing isolation must be determined individually. Decisions on the need for treatment as an inpatient must be taken individually, taking into account factors such as the severity of the tuberculosis, underlying diseases or comorbidity, problems with therapy and patient cooperation. If the appropriate framework conditions are in place (medical care, patient reliability, home circumstances), clinically stable tuberculosis patients can be treated as outpatients [37].

### Therapy adherence

The most important prerequisite for treatment to succeed is reliable, regular intake of anti-tuberculosis drugs. This must be properly explained to patients. They must be told in understandable terms why tuberculosis must be treated with a combination of drugs for such a long time. If reliable intake of medication is not assured or is in doubt, the treatment must be supervised directly (DOT = directly observed treatment). As a matter of principle, medicines must be taken under supervision during a stay in hospital.

The obligation under the German Prevention and Control of Infectious Diseases Act (Infektionsschutzgesetz, IfSG) to notify refusal or premature withdrawal from treatment is intended to facilitate the identification of patients in need of active support and possibly also close supervision during their treatment.

### General hygiene

Patients must be encouraged to adopt hygienic behaviour (coughing into a paper handkerchief, turning away from other people) and to wear a mask over mouth and nose. In a recent study, it was shown for the first time that the MTB concentration in the air of the patient’s room is lower if the patient wears a mask [38]. When contamination of the hands with pathogen-containing material is to be feared (e.g. during oral hygiene, aspiration, catheter maintenance and urine disposal in tuberculosis of the urinary tract, and bandage changing or wound dressing when skin secretions contain pathogens), personnel must wear protective gloves and, in the event of aerosol-generating procedures, a respirator mask (see below). Personnel at risk, fellow patients and other contact persons must be given detailed, understandable instructions about possible paths of infection and the necessary protective measures.

### Ventilation

Adequate ventilation of rooms where infectious tuberculosis patients are accommodated is of crucial importance, as it helps to dilute or eliminate infectious aerosols and minimise the further spread of germs into the environment (no recirculation of air containing bacteria, air flow towards the patient’s room and from there to outside) [34,36,39]. If a ventilating and air-conditioning system (with negative air pressure in the isolation room and positive air pressure in the anteroom) is used, there should be a change of air at least four to six times per hour. Some authors call for a frequency of nine to twelve times an hour. No scientific arguments could be found for a precise minimum frequency of air change [35].

### Respiratory protection

There is no evidence-based proof of the effectiveness of respiratory masks in preventing *M. tuberculosis* infection. Given that diagnosis, therapy, and the control and infection prevention measures outlined above already significantly reduce the risk of transmission via known or suspected tuberculosis cases, respiratory masks make a limited contribution toward risk reduction. However, where there is exposure to higher concentrations of aerosols, it makes sense to wear one.

Like the masks worn in industry, better fitting, particle-filtering half masks with improved filtration are available for medical needs (filtering face piece = FFP). The FFPs currently available are classified according to European standards (EN 149). The total leakage from a mask is made up of leakages around the face, leakage around the exhalation valve (if there is one) and leakage from the actual filter penetration. The average total leakage for Class 1 respiratory masks must not exceed 22% under test conditions. That of Class 2 masks must not exceed 8% and Class 3 must not exceed 2% (with an average particle diameter of 0.6 μm). FFP2 masks (or equivalent) are recommended as a personal protective measure for preventing infection in case of tuberculosis. The wearer must be instructed and the fit must be checked, as the protective effect of the respirator can only be reliable if it fits properly and is used correctly [40].

In the presence of other persons, patients should wear at least conventional mouth and nose covering masks inside and always outside the isolation room so as to reduce the spread of aerosol (see above).

In order to ascertain the need for personnel to use respiratory masks, a risk analysis should be undertaken for the relevant institution or relevant department and the specific patient. FFP2 masks are used either where exposure to the coughing of patients suffering from suspected or confirmed open tuberculosis is unavoidable, or where a high concentration of aerosol must be assumed. In special situations, a specific risk assessment must be carried out to identify suitable respiratory protection.

### Disinfection and sterilisation

Thermal disinfection is always preferable to chemical disinfection, as it is safer, more reliable (inter alia because of the possibility of automation) and less harmful (allergies resulting from chemical disinfectants, environmental pollution). Industry has developed devices for different fields of medicine that enable thermal disinfection of a variety of instruments, respiratory accessories, etc. [37].

Surfaces should be disinfected by standard hygienic methods. Spray disinfection is not sufficiently effective. Although microorganisms, and thus also tuberculosis bacteria, can be detected on surfaces such as walls, floors and furniture, they are very rarely a real infection risk for humans because no aerosols are formed.

Special disinfection of urine or stool is not necessary. If pathogen-containing excretions are collected, they can be disposed of by thermal disinfection (with customary cleaning and disinfection equipment).

Disinfectant cleaning of surfaces that may be contaminated with pathogen-containing particles should also be carried out in the home. Clothing should be washed at a minimum of 60°C with customary laundry detergents and carpets should be cleaned to remove dust particles as far as possible, if need be by spray extraction. The spraying of formaldehyde is no longer permitted.

### Precautions against tuberculosis

Contact tracings by health authorities pursuant to IfSG serve to identify at an early stage cases of TB and individuals who can benefit from preventive chemotherapy. TB screening by occupational health physicians serves the same purpose.

As the TB incidence in Germany decreased over recent decades (Figure 1), in order to avoid unnecessary X-ray examinations due to a false positive TB test there was a paradigm shift to optional checks as warranted by accidental occupational exposure to *M. tuberculosis*. According to the German regulation on occupational healthcare provisions (ArbMedVV), employees must be offered tests ‘if as a result of exposure to biological agents a serious infection or disease can be expected and post-exposure prophylactic (prevention) measures are possible’. This applies to TB.

In contrast, obligatory checks pursuant to the ArbMedVV are only provided for in the case of employees in tuberculosis departments and other pneumological facilities or research institutes and laboratories who have regular contact with infectious patients or materials. If regular obligatory checks are performed, there is no need for an additional test after unprotected contact with an infectious patient or infectious materials.

Participation in an optional check or obligatory occupational health check in accordance with ArbMedVV replaces contact investigations pursuant to IfSG. In order to avoid duplicate tests, it makes sense for occupational health physicians to conduct their tests in consultation with public health authorities and to use the appropriate formula for checks as per the German Central Committee to Combat Tuberculosis (Deutsches Zentralkomitee zur Bekämpfung der Tuberkulose, DZK) recommendation for contact investigations [5]. If an employee fails take up the option of a test, the local public health authority is responsible for performing the contact investigation in accordance with IfSG.

The aim of TB screenings is to identify employees with a recent LTBI, as they will benefit most from preventive chemotherapy. It is therefore not expedient to examine all possible contact persons. Instead, only ‘close contact persons’ should be tested, as the proportion of recent infections among them will be higher in close contacts.

The probability of transmission depends on the patient’s infectiousness (microscopically positive or negative), proximity to the infectious source, and the duration of contact. This suggests the following criteria for selecting contact persons [5]:

A) brief but intensive contact with a coughing, sputum-positive patient without protection,

B) cumulative contact duration of at least eight hours with a patient with microscopic evidence of AFB in a direct specimen of sputum,

C) cumulative contact duration of at least 40 hours with a patient with only culturally or molecular biologically confirmed evidence of tuberculosis.

In practice, this rule is often difficult to apply because the contact duration cannot be reliably estimated or because uncertainty among employees may lead them to want a check-up. Hence the above-mentioned criteria should be seen only as guides to the selection of contact persons.

In the case of optional checks, an IGRA should be carried out only eight weeks after the last contact with the index patient or the infectious materials, so as to leave sufficient time for the immunological reaction to develop. A chest X-ray after a positive IGRA should be done only three months after the last contact with the possible source of infection, since development of active TB is unlikely before then. The flow chart below is based on the recommendations for contact investigations [5].

Preventive check-ups in accordance with ArbMedVV are repeated regularly (obligatory test) or as warranted after unprotected contact. This raises the question how different IGRA results in serial tests are to be interpreted. The variability of IGRA in serial tests is not yet sufficiently understood [6,13,41]. Reversion of positive IGRA results into negative results is more frequent than conversion of negative IGRA results into positive ones. More significant is the fact that the probability of conversion or reversion depends on the quantitative test results of the first IGRA. Thus a grey area could be helpful in separating genuine conversions and reversions and chance deviations. The Center for Disease Control and Prevention (CDC) and the European Centre for Disease Prevention and Control have proposed a grey area in the range 5–7 spot forming cells (SFC) for T-SPOT.TB [42]. As yet, however, no consensus has been reached on a grey area for QuantiFERON-TB Gold In-Tube (QFT). A grey area of 0.2 and 0.7 IU/mL and defining conversion and reversion as overstepping this grey area minimise the rate of conversion and reversion and keep the proportion of employees whose QFT values are within this grey area relatively low [43-45].

Since the reversion rate with IGRA is higher than expected [6,13], ‘once positive, always positive’, the statement that used to apply to TST, is obsolete. All healthcare workers should be re-tested with the IGRA in a planned routine screening. This reduces the number of chest X-rays needed, since no further medical check is necessary if the IGRA reverts to negative and there are no recognisable clinical signs of active TB. Chemoprevention (see below) in employees with fluctuating test results requires further discussion. Assuming that recent exposure to *M. tuberculosis* is probable, the simplest approach would be to rule out lung tuberculosis if the IGRA result is positive and not to carry out any further medical examination if the test result is negative and there are no recognisable clinical symptoms of tuberculosis. The same applies to employees with results in the grey area. If they have no clinical symptoms, no further medical examination is necessary. However, more data on progression to the disease is needed in order to deduce evidence-based recommendations for chemotherapy treatment for employees with an IGRA conversion or reversion, and for employees with IGRA results in the grey area.

**Figure 3:**
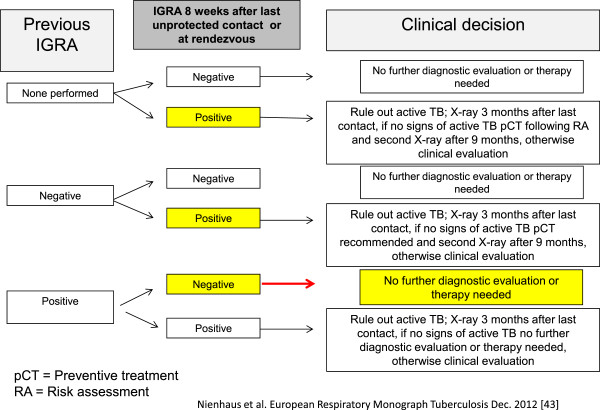
**Decision tree for repeated IGRA according to **[46]**.**

Employees whose anamnesis includes chemotherapy for LTBI or those with repeat positive IGRA results should not be re-tested in subsequent preventive checks since the IGRA results would not be revealing. If one of these employees is part of a cohort with a low risk of progression (e.g. no active tuberculosis among hospital personnel in recent years), a chest X-ray should only be taken if there are suspicious clinical symptoms. Figure 3 shows a decision tree for repeat preventive checks for healthcare workers in accordance with ArbMedVV. However, it should be noted that further research is needed to prove the usefulness of this approach in countries with low TB incidence and high hygiene standards [46]. Furthermore it needs to be pointed out that this recommendation seems helpful for countries like Germany with currently low TB incidence but history of high TB incidence and high prevalence of BCG vaccination in HCWs. In countries with a long history of low TB incidence and no BCG vaccination policy and therefore low rates of positive TST results in HCWs other conclusions might be drawn as is shown in the Canadian recommendations. In Canada for serial testing of HCWs the TST is recommended because compared to TST unexplained variability of the IGRA seems to be higher [47,48].

### Chemoprevention

The probability of developing active TB during the first two years following a positive IGRA is significantly lower for healthcare workers than for close contacts in the general population [10,11]. This can probably be explained by the fact that healthcare workers do not have the typical risk factors for developing active TB (alcoholism, homelessness, etc.) and that a positive IGRA in healthcare workers is often caused by an remote LTBI with a low risk of progression. This should be taken into account when selecting individuals to be treated with preventive chemotherapy. Chemoprevention should only be considered if the employee is under 50 and the IGRA is positive after recent exposure to *M. tuberculosis*[5].

Before initiating chemoprevention, a chest X-ray must be taken to rule out active TB and a further check must be carried out after completing chemoprevention so as not to overlook a disease that may be developing despite chemoprevention.

Chemoprevention involves taking Isoniazid (INH) 300 mg daily for six or preferably nine months. This therapy for the treatment of LTBI can achieve 90% protection [49]. In practice, however, effectiveness is significantly lower, mainly because of insufficiently regular intake of the medication for this long period.

If the MTB of the index case is resistant to INH, or the contact (i.e. the HCW) is unable to tolerate INH, as an alternative 600 mg Rifampicin (RMP) can be taken for four months, or a combination of INH and RMP for three to four months for chemoprevention [50]. However, the effectiveness of these two alternatives has been less well researched.

### Hepatotoxicity of chemoprevention with INH

Acceptance of chemoprevention is reduced by the fear of possible liver damage. Until now, only two meta-analyses have dealt with the number of INH-induced hepatitis in adults. According to these analyses, the probability of INH-associated hepatitis is 0.6%. Occasionally, however, significantly higher rates of up to 5% were described [49,51]. Because of the hepatotoxic side effect of INH, regular laboratory controls (after two and four weeks, then monthly) are necessary. If there is a significantly raised level of liver enzymes, chemoprevention should be discontinued.

### New treatment regime in chemotherapy

Significantly shorter treatment periods and considerably fewer tablets than with previous offers are the key elements for improving the results of preventive treatment in persons with freshly diagnosed LTBI. Two recently published, extensive and randomly controlled studies provide evidence that the combined therapy of INH and Rifapentin (RPT) administered once a week for twelve weeks is less toxic than classic INH therapy and equally effective [52,53]. In the United States, therefore, the CDC recommends this combination therapy [54].

Should the new regime become established, this will make it considerably easier for occupational health physicians to advise chemoprevention and further reduce the number of healthcare workers requiring treatment for TB at a later date.

### Tuberculosis as an occupational disease in Germany

Unlike in other European countries such as France [55], it is not generally assumed in Germany that healthcare workers with patient contact run a greater risk of TB infection, and therefore of contracting the disease. Therefore, according to number 3101 of the German Ordinance on Occupational Diseases (Berufskrankheitenverordnung, BKV), tuberculosis in a healthcare worker can only be recognised as an occupational disease if an increased occupational risk of infection can be proven and if there is no (competing) risk of infection outside work [56]. According to BKV, both tuberculosis in HCWs and silico-tuberculosis in workers exposed to silica dust can be recognised as occupational diseases. Furthermore, active TB as the result of an accident can be compensated by statutory accident insurance schemes. In addition to work postings to countries with a high incidence of TB, this is also relevant in the case of occupational exposure to *M. tuberculosis*, i.e. contact to a college with active TB outside the healthcare sector. Transmission of M. tuberculosis can be considered an accident as it takes place within 24 hours, the juridical definition for the relationship of cause and effect in the realm of the social accident insurance legislation in Germany.

As shown in Table 3, in addition to close contact with a known index person (infectious patient), healthcare workers run an increased risk of infection from work in specialist lung clinics and laboratories (Category A) if they regularly come into contact with tuberculosis patients or sputum samples. However, a raised risk of infection can also be explained epidemiologically (Category B) [25,31].

**Table 3:** **Activities categorised by infection risk according to**[51]

**Category A**	**Category B**	**Category C**	**Category D**
Activity, Area			
TB ward, specialist lung clinic, specialist lung doctors, micro-biology laboratories that examine sputum	Bronchoscopies, laryngoscopy, emergency intubation, post mortems, work in infection wards, emergency services, A & E, geriatrics and geriatric care*, looking after at-risk groups, deployment abroad in areas with a high incidence	General hospitals	All other health service and welfare work
		General practitioners	
		Dental practices	
Easing of burden of proof			
Yes, index not necessary	Yes, index not necessary	No, index necessary, exceptions are possible	Index necessary
Reasons			
Infection risk due to particular patients or materials	Infection risk proven by epidemiological studies	Infection risk insufficiently proven by epidemiological studies. Index may be dispensed with if there are several patients with open TB in the work area.	Infection risk not proven by epidemiological studies

*If there is close contact with patients in need of care.

There is a special risk from unknown, or belatedly recognised, tuberculosis. Often, but not necessarily, Category B activities are associated with close contact with the air the patient exhales (bronchoscopy, intubation, extubation, etc.). An increased risk of infection may also arise if once in a while tuberculosis patients are diagnosed and treated in a particular area (Category C). Even if it is out of the question that the known patient is the index person, e.g. because there was no personal contact with the particular patient, in such institutions one can expect the rate of unrecognised TB cases in this group of patients to be higher than in the population at large. Typical institutions are e.g. general practices and general hospitals without infection wards.

Category D includes institutions where there is no indication of an increased rate of tuberculosis among patients. This category includes all institutions that do not fall under Category A to C, such as dermatologists, surgeons (except chest surgeons), rehab clinics, psychotherapy practices and physiotherapists (as long as they do not carry out respiration therapy).

In assessing the circumstances in occupational disease claim proceedings there is no need to look for an index person in Categories A and B. Instead, the typical clientele of patients (Category A) or epidemiological data (Category B) make it easier to find proof. In Category C it is possible to recognise the causal links even if no index person could be found. However, judgement must be passed on a case-by-case basis. No general statement can be made as to the number of TB cases per year from which there is an increased risk of infection. In Category D, recognition as an occupational disease is only possible if a plausible index person can be identified.

**Figure 4:**
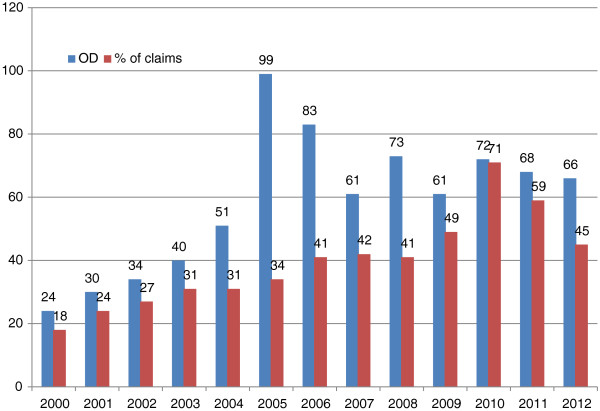
**Number of active TB cases compensated as occupational disease (OD) and percentage of recognized claims in the years 2000 to 2012 according to **[1]**.**

Since the publication of the new recognition criteria for the first time in 2003 [56], there has been a rise both in the number of tuberculosis cases reported and in the number recognised as occupational diseases at the Institute for Statutory Accident Insurance and Prevention in the Health and Welfare Services (Berufsgenossenschaft für Gesundheitsdienst und Wohlfahrtspflege, BGW) (Figure 4). In 2000, 24 HCWs with tuberculosis were recognised as having an occupational disease. In 2012, the number was 66. The rate of recognition among the decided claims rose from 18% in 2000 to 45% in 2012.

Since the risk of infection in the healthcare sector has not risen in recent years, one can assume that the increase in tuberculosis as an occupational disease is attributable to the extension of criteria that ease the burden of proof. An analysis of 150 expert opinions in claim proceedings cases found that it had been recommended that the burden of proof be eased in every second case (52%). Easing the burden of proof has halved the average time taken by occupational disease proceedings in Germany [56].

**Figure 5:**
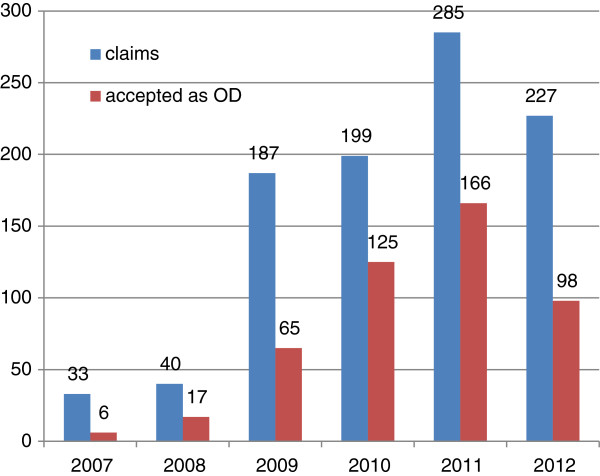
**Number of latent tuberculosis infections (LTBIs) reported and recognised as occupational diseases in the years 2007 to 2012 at BGW according to **[1]**.**

In addition to active tuberculosis, an LTBI can be reported as an occupational disease. The number of LTBIs reported as occupational diseases has risen significantly in the last few years (Figure 5). This increase, too, cannot be explained by an increase in the risk of infection in recent years. Rather, the increased reporting is based on the fact that for some years the above-mentioned IGRA has rendered the diagnosis of LTBI more valid in a population of HCWs with a high prevalence of BCG vaccination. It makes sense to report LTBI as an occupational disease especially if preventive chemotherapy is being considered. Preventive chemotherapy makes sense if a young healthcare worker (e.g. below the age of 35) has a positive IGRA eight weeks after close contact with a microscopically positive patient (see above). In this case, it is probable that the cause of the LTBI was occupational. There is no need for laborious investigations in occupational disease claim proceedings and the costs of consultation with and treatment by a pneumologist can be paid by the statutory accident insurance scheme under Paragraph 3 (Prevention) of the Ordinance on Occupational Diseases (BKV).

## Conclusion

TB in HCW and nosocomial transmission of TB will remain a challenge for OSH experts as the downward trend in TB incidence in the general population has seemingly come to a halt and the TB incidence in migrants is still high in Germany. Furthermore, the pool of LTBI in HCW is large, especially in those who started work during the decades when TB incidence in Germany was higher. IGRAs allow for a better assessment of the infection risk than TSTs. However, they do not permit a distinction between remote and recent infection. Therefore evaluation of recent exposure remains important when choosing the target group for preventive treatment. Variability of IGRA results is not jet well understood and we need epidemiologic studies in order to decide the most effective and efficient screening strategy in HCWs.

## Competing interest

RD received a travel grant from Qaigen/Cellestis and Oxford Immunotech and an unrestricted research fund from Qaigen/Cellestis. All other authors declare that they do not have any direct or indirect personal relationship, affiliation or association with any party with whom they deal in their day-to-day work that would give rise to any actual or perceived competing interest.

## Authors’ contributions

AN drafted the first version of the manuscript. AS, AMP, FCR and RD made substantial suggestions and comments for revision of the first draft. All authors read and approved the final version of the manuscript.
